# Sick leave and disability pension among TMD patients with musculoskeletal diseases, mental and behavioural disorders – a SWEREG-TMD population-based cohort study

**DOI:** 10.1186/s12889-023-15815-4

**Published:** 2023-05-11

**Authors:** Adrian Salinas Fredricson, Carina Krüger Weiner, Johanna Adami, Annika Rosén, Bodil Lund, Britt Hedenberg-Magnusson, Lars Fredriksson, Pia Svedberg, Aron Naimi-Akbar

**Affiliations:** 1 Public Dental Services, Folktandvården Stockholm, Eastmaninstitutet, Department of Oral and Maxillofacial Surgery, Eastmaninstitutet Käkkirurgi, Dalagatan 11, 102 31 Stockholm, Sweden; 2 Division of Oral Diagnostics and Rehabilitation, Department of Dental Medicine, Karolinska Institutet, Stockholm, Sweden; 3 Department of Oral and Maxillofacial Surgery, Gävle County Hospital, Gävle, Sweden; 4 Sophiahemmet University, Stockholm, Sweden; 5 Department of Clinical Dentistry, Division of Oral and Maxillofacial Surgery, University of Bergen, Bergen, Norway; 6 Department of Oral and Maxillofacial Surgery, Haukeland University Hospital, Bergen, Norway; 7 Medical Unit for Reconstructive Plastic- and Craniofacial Surgery, Karolinska University Hospital, Stockholm, Sweden; 8 Department of Orofacial Pain and Jaw Function, Public Dental Services, Folktandvården Stockholm, EastmaninstitutetStockholm, Sweden; 9 Division of Insurance Medicine, Department of Clinical Neuroscience, Karolinska Institutet, Stockholm, Sweden; 10 Health Technology Assessment-Odontology (HTA-O), Malmö University, Malmö, Sweden

**Keywords:** Temporomandibular disorder, Sick leave, Disability pension, Registry-based research, TMJ surgery, Cohort study, Comorbidities, Musculoskeletal diseases, Mental and behavioural disorders

## Abstract

**Background:**

Temporomandibular disorders (TMD) are associated with musculoskeletal diseases (MSD), mental and behavioural disorders (MBD), and patients with TMD have been shown to have 2–3 times more days of sick leave (SL) and disability pension (DP) than the general population. MSD and MBD are two of the most common causes for SL and DP, and the association between TMD and the influence of comorbidities on the need for SL and DP among TMD patients need further clarification. This study investigates the impact of MSD and MBD comorbidity on SL and DP among TMD patients diagnosed in a hospital setting and/or surgically treated.

**Methods:**

All incident TMD patients diagnosed or treated in a hospital setting between 1998 and 2016 and aged 23–59 were included. A non-exposed comparison cohort was collected from the general population. The cohorts were grouped based on the presence of comorbidity: No comorbidity (Group I); MSD comorbidity (Group II); MBD comorbidity (Group III); and combined MSD and MBD comorbidity (Group IV). Main outcomes were mean annual days of SL and DP, and statistical analysis was conducted using generalized estimated equations.

**Results:**

TMD subjects with no comorbidities (Group I) and with MSD/MBD comorbidity (Group II and III) were 2–3 times more often on SL and DP than the corresponding groups from the general population. However, in the group with both MSD and MBD comorbidity (Group IV), the difference between the TMD subjects and the general population was diminishing, suggesting an additive effect.

**Conclusion:**

TMD patients are more dependent on SL and DP benefits compared to general population and the difference remains even after considering MSD and MBD comorbidity. In individuals with combined MSD and MBD comorbidity, concurrent TMD has less impact on the need for social insurance benefits. The results accentuate the impact TMD has on the patients’ impaired ability to return to work and why TMD should be recognized as having a substantial impact on individual and economic suffering as well as on societal costs, with emphasis on the influence of comorbidities on patient suffering.

## Introduction

Temporomandibular disorder (TMD) is associated with orofacial pain, dysfunction, and impaired quality of life and has an incidence rate of 4% in the general population [1, 2]. TMD is also associated with several comorbidities such as diseases of the musculoskeletal system and connective tissue (MSD) and mental health and behavioural disorders (MBD) [3–5].

Globally, the leading causes of years lived with disability (YLD) are MBD, MSD, diabetes, chronic obstructive pulmonary disease and endocrine diseases [6]. Low back pain, headache disorders, depressive disorders, anxiety disorders and diabetes have been the leading cause for YLD during the last 30 years in countries with high sociodemographic index (SDI). YLD rates are globally highest among elderly but for working-age individuals the pattern of leading causes for YLD prevails, with MBD, MSD and neurological disorders accounting for 45% of all YLD among individuals aged 20–54 [7]. As MSD and MBD result in many years of heavy disease burden, it is not surprising that these conditions also lead to an increased dependence on social insurance benefits, such as sick leave (SL) and  disability pension (DP). A report by the Swedish Social Insurance Agency showed that during 2020, MSD (ICD M00-M99) was the second most common cause for SL/DP, accounting for 15/18% (F/M) of Sweden’s SL costs and 12/8% (F/M) of all DP costs [8]. The most common cause was MBD (ICD F00–F99), which accounted for 49/38% (F/M) of all SL costs and 87/88% (F/M) of all DP costs in 2020. This has been the trend over the last 20 years with a gradual increase of MBD costs in comparison to MSD, which was the leading cause of both SL and DP during the early 2000s [8]. Factors such as sex, profession, socioeconomic status, living conditions, and living area also impact the need of both SL and DP [9–11].

An earlier study from SWEREG-TMD has shown that TMD has a great impact the patients’ need for both SL and DP, with mean annual days of SL and DP two to three times higher than the general population [12]. The close association between TMD, MSD and MBD and these diagnoses' impact on social security benefits raises important questions, and the impact of other diagnoses on SL and DP among TMD patients needs clarification. Therefore, this study used the Swedish Registry Studies for Surgically Treated TMD (SWEREG-TMD), which is based on registry data prospectively collected from various Swedish national registries, to investigate the reliance on social insurance benefits among TMD patients with comorbidities.

## Materials and methods

### Study design and data sources

The details of this registry-based cohort study have been described earlier in previous publications from the SWEREG-TMD [4, 5, 12]. Three nationwide registries administrated by the National Board of Health and Welfare (NBHW) and Statistics Sweden (SCB) were used in this study.

1. National Board of Health and Welfare (NBHW)
  a The National Patient Registry (NPR) was used to collect the data on incident TMD or surgical treatment from 1998–2016. The NPR was also used to collect MSD and MBD comorbidity from 1964 to the time of inclusion. The NPR is administered by the NBHW and includes 100% of all in-patient care and 80–100% of all out-patient visits, with information on both primary diagnosis and procedure codes. The positive predictive value of both in- and outpatient care is 85–95% [13].
  b The Total Population Registry (TPR) was used to collect the unexposed comparison cohort [14]. The TPR has existed since 1968 and has a 100% coverage of Swedish citizens’ births and deaths.
2. Statistics Sweden (SCB)
  a The Longitudinal Integrated Database for Health Insurance and Labour Market Studies (LISA) was used to collect information on SL, DP, and sociodemographic covariates. SL and DP is prospectively registered on an annual basis [15].

Linking individuals between the registries was made possible through the use of the Personal Identification Number (PIN) [16]. NBHW was responsible for linking and collecting the cohorts, exposures, outcomes, and comorbidities before delivering the pseudo anonymized data sets with constructed identification numbers to the research group.

### Exposures

Main exposure was collected from the NPR and defined as first registered TMD diagnosis (K07.6) and/or surgical treatment for TMD in a hospital setting between 1998 and 2016. First time of diagnosis or surgical treatment was considered as T0, meaning T0 could be any year during 1998–2016. Surgical treatment codes used as proxy for TMD were TMJ disc surgical procedure (EGB10), TMJ arthroscopy (EGA00), TMJ condylectomy (EGB00), TMJ prosthesis surgical procedure (EGC30), other surgical reconstruction of TMJ (EGC99), TMJ synovectomy (EGB20), TMJ biopsy (EGA20), injection of diagnostic or therapeutic substance in the TMJ (TEG10), TMJ condylotomy (EDC00), open reposition of TMJ luxation (EGC00), TMJ plastic surgery (EGC10), and TMJ plastic surgery with bone graft or other type of transplant (EGC20). The exposed cohort was divided into two sub-cohorts: non-surgical (NS) (TMD diagnosis without subsequent surgical treatment) and surgical (S) (at least one surgical intervention).

The non-exposed (NE) comparison cohort was collected from the TPR and matched 1:10 for age, sex, and living area with the requirement of being alive at the time of inclusion. As working-age in Sweden typically spans from 18–65, only exposed and non-exposed subjects aged 23–59 at the time of inclusion were included in this study, to allow subjects to be monitored during the entire 10-year follow-up time.

Comorbidities were previous diagnoses of MSD and/or MBD recorded in the NPR before the time of inclusion. ICD-10 codes were used to identify both MSD (M00–M99) and MBD (F00–F99) comorbidity. Data on comorbidity were collected from 1964 up to T0, i.e., all comorbidities were collected before first TMD-diagnosis or surgical treatment. ICD-10 was introduced in Sweden in 1997, and older versions of ICD-codes – i.e., ICD-7 (1958–1968), ICD-8 (1969–1986), and ICD-9 (1987–1996) – were translated to ICD-10 codes in accordance with instructions and a codebook provided by the NBHW.

MSD comorbidity was defined as diagnoses within the 13^th^ chapter of ICD-10 (M00–M99), and MBD was defined as diagnoses within the 5^th^ chapter of ICD-10 (F00-F99). The division into diagnostic blocks are described in Table 1.

**Table 1:** Listing of included MSD and MBD diagnoses and the corresponding ICD-10 codes and blocks

ICD-10	Block
Diseases of the musculoskeletal system and connective tissue	
M00-M25	Arthropathies
M30-M36	Systemic connective tissue disorders
M40-M54	Dorsopathies
M60-M79	Soft tissue disorders
M80-M94	Osteopathies and chondropathies
M95-M99	Other disorders of the musculoskeletal system and connective tissue
Mental and behavioural disorders	
F00-F09	Organic, including symptomatic, mental disorders
F10-F19	Mental and behavioural disorders due to psychoactive substance use
F20-F29	Schizophrenia, schizotypal and delusional disorders
F30-F39	Mood affective disorders
F40-F48	Neurotic, stress-related and somatoform disorders
F50-F59	Behavioural syndromes associated with physiological disturbances and physical factors
F60-F69	Disorders of adult personality and behaviour
F70-F79	Mental retardation
F80-F89	Disorders of psychological development
F90-F98	Behavioural and emotional disorders with onset usually occurring in childhood and adolescence
F99	Unspecified mental disorder

To stratify on comorbidity, the following four groups of comorbidity presence were constructed:

- Group I: No comorbidity present,
- Group II: MSD comorbidity present, but no MBD comorbidity,
- Group III: MBD comorbidity present, but no MSD comorbidity, and
- Group IV: Both MSD and MBD comorbidity present.

### Outcomes

Outcome variables were SL and DP, collected from LISA between 1994 and 2017. SL is available for all Swedish citizens from the age of 16, and the first 14 days are reimbursed by the employer. DP is available for individuals aged 19–64 with lifelong incapacity of work. Both SL and DP are awarded in levels of 25, 50, 75 or 100% For both outcomes, net days were used – i.e., partial reimbursements recounted into full annual days. Only SL spells lasting more than 14 days were included since only those are included in the LISA registry. In their current forms, SL and DP are available in the LISA-registry from 1994.

### Covariates

Covariates adjusted for in the model were: sex, level of education (0–9 years, 10–12 years, and > 12 years), country of birth (Sweden, Other Nordic countries, European countries, and Non-European countries), living area according to Eurostat’s Degree of Urbanisation (DEGURBA) (cities, towns, and rural), and age (23–25, 26–30, 31–35, 36–40, 41–45, 46–50, 51–55, and ≥ 56). Calendar year was also adjusted for, due to fluctuations in the policies of SL and DP insurance.

### Follow-up time

**Fig. 1:**
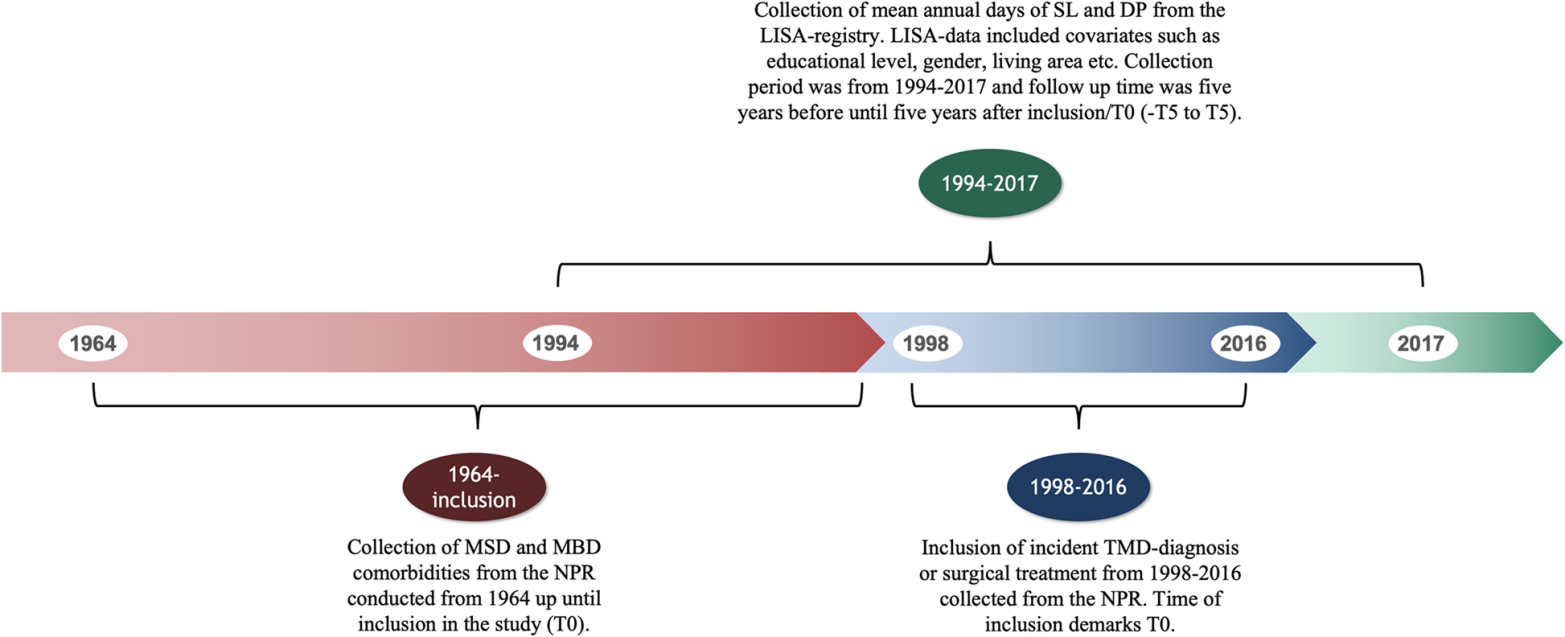
Timeline over the period of inclusion and the collection of comorbidities and outcomes. DP: Disability pension, LISA: Longitudinal Integrated Database for Health Insurance and Labour Market Studies, MBD: Mental and behavioural disorders, MSD: Diseases of the musculoskeletal and connective tissue, NPR: National Patient Registry, SL: Sick leave, -T5: Five years before inclusion, T0: Year of inclusion, T5: Five years after inclusion, TMD: Temporomandibular disorders

The period of inclusion was 1998–2016, and the year of inclusion was defined as T0. Follow-up time for the outcomes SL and DP was five years before T0 (-T5) up to five years after T0 (T5). The subjects’ individual follow-up time of 10 years was included in the regression model. The follow-up time, the period of inclusion and the collection of comorbidities and outcome is depicted in Fig. 1.

### Statistical methods

All analyses were conducted using Stata v.16.1 software (Stata Corporation LLC, College Station, USA). Generalized estimating equations (GEE) was used to analyse number of net days on SL and DP during -T5 to T5 [17]. SL and DP were modelled as repeated measurements assuming Poisson distribution. The regression model was stratified on presence of comorbidity (Group I–IV) and adjusted for educational level, age, sex, birth country, DEGURBA, and calendar year. Missing values were imputed using Multiple Imputation by Chained Equations (MICE), assuming missing at random with 20 imputations for each missing [18]. Multinominal logistic regression (DEGURBA and country of birth) and ordinal logistic regression (educational level) were used to impute missing data.

## Results

### Recruitment

**Fig. 2:**
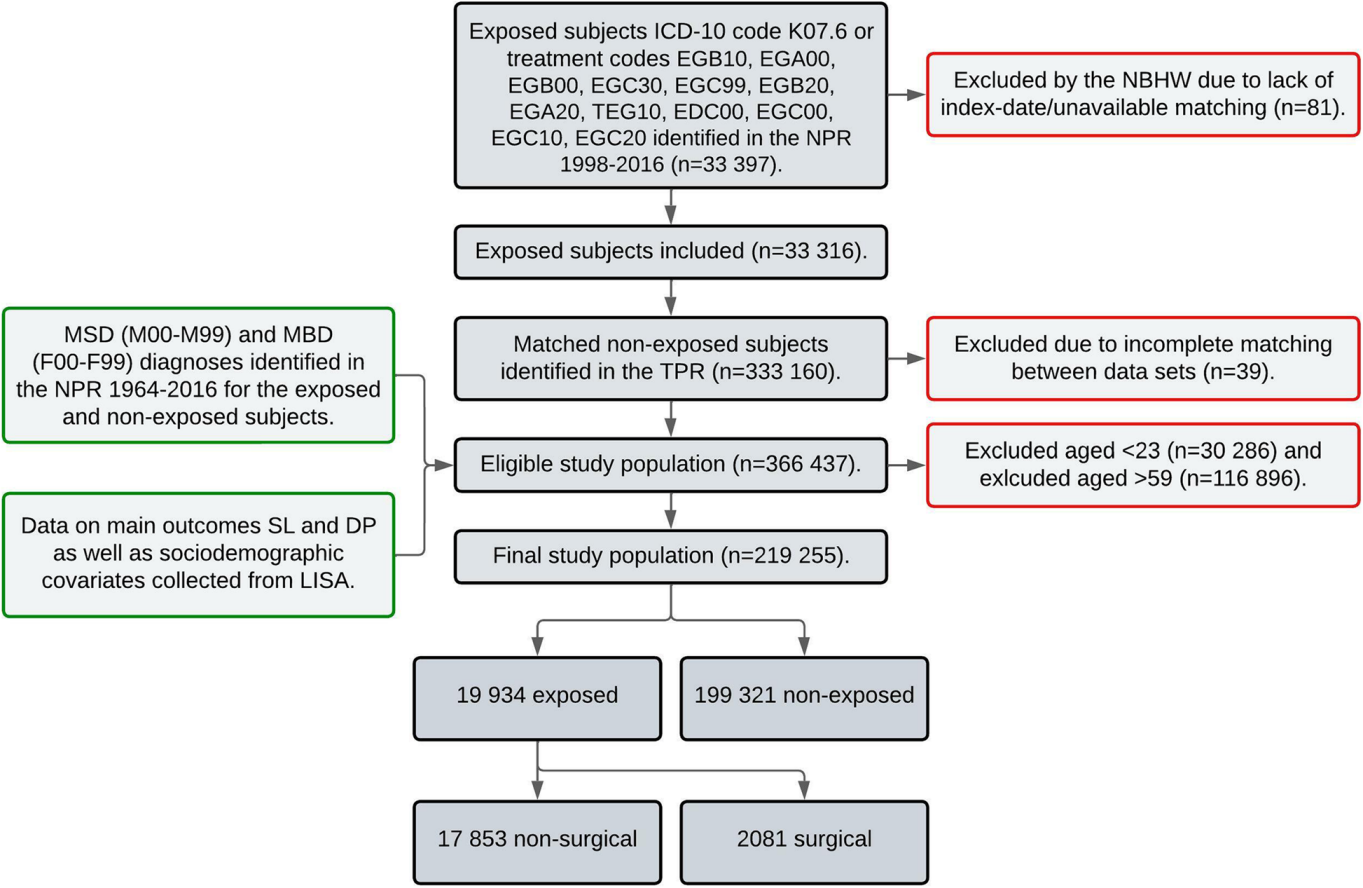
Flow chart of the recruitment 1998–2016 with descriptions of the registries used to collect the outcome variables SL and DP as well as exposure variables and covariates. The figure is a modified version of earlier presented data. ICD-10: International Classification of Diseases (10^th^ revision). LISA: Longitudinal Integrated Database for Health Insurance and Labour Market Studies, MBD: Mental and behavioural disorders, MSD: Diseases of the musculoskeletal and connective tissue, NBHW: National Board of Health and Welfare, NPR: National Patient Registry, TPR: Total Population Registry

Figure 2 illustrates the recruitment process of the cohorts as well as the collection of outcomes, exposures, and covariates from respective registries. The figure is a modified version of earlier presented data from SWEREG-TMD [4, 5, 12].

### Descriptive statistics

The sociodemographic baseline data of the study sample as well as the exposure of comorbidities are presented in Table 2. In the TMD cohort, there was a sex difference: 71.8% of the women in the non-surgical cohort and 78.8% in the surgical cohort. The surgical cohort had higher percentage of subjects born in Sweden and living in cities while a lower mean age. The largest differences between the TMD cohorts and the general population were found in relation to comorbidities, where almost 70% of the general population cohort had no comorbidities recorded while the corresponding numbers for the TMD cohorts were 49.9% (non-surgical) and 46.4% (surgical). Table 2 depicts specific comorbidities recorded, where the differences in prior MSD (M00–M99) between the non-exposed cohort and the surgical cohort were 22.7% and difference in prior MBD (F00–F99) was 7.5%.

**Table 2:** Baseline data of the cohorts including sociodemographic information, sex, comorbidities, and Groups I–IV

	**General population cohort**	**Exposed cohort**	
		Non-surgical	Surgical
	n = 199 321 (%)	n = 17 853 (%)	n = 2081 (%)
**Sex**			
Female	144 542 (72.5)	12 817 (71.8)	1639 (78.8)
Male	54 779 (27.5)	5036 (28.2)	442 (21.2)
**Educational level**			
0–9 years	23 555 (11.8)	2330 (13.1)	273 (13.1)
10–12 years	84 940 (42.6)	8003 (44.8)	986 (47.4)
> 12 years	88 504 (44.4)	7393 (41.4)	818 (39.3)
Data unavailable	2322 (1.2)	127 (0.7%)	4 (0.2)
**Country of birth**			
Sweden	154 467 (77.5)	12 845 (72.0)	1774 (85.3)
Other Nordic countries	5558 (2.8)	465 (2.6)	75 (3.6)
Other European countries	16 120 (8.1)	1512 (8.5)	75 (3.6)
Non-European countries	23 153 (11.6)	3030 (17.0)	157 (7.5)
Data unavailable	23 (< 0.1)	1 (< 0.1)	0 (0)
**Degree of urbanisation**			
Cities	96 433 (48.4)	8319 (46.6)	1096 (52.7)
Towns and suburbs	51 885 (26.0)	5210 (29.2)	511 (24.6)
Rural areas	50 044 (25.1)	4283 (24.0)	469 (22.5)
Data unavailable	959 (0.5)	41 (0.2)	5 (0.2)
**Age**			
Mean	41.63	41.87	39.62
Median	42	42	39
Range	23–59	23–59	23–59
**Marital status**			
Married	86 937 (43.6)	8128 (45.5)	849 (40.8)
Not married	82 317 (41.3)	6886 (38.6)	933 (44.8)
Divorced	27 038 (13.6)	2629 (14.7)	276 (13.3)
Widow/widower	1869 (0.9)	145 (0.8)	14 (0.7)
Other^a^	201 (< 0.1)	24 (< 0.1)	4 (< 0.1)
Data unavailable	959 (0.5)	41 (0.2)	5 (0.2)
**Group**			
Group I	136 372 (68.4)	8911 (49.9)	965 (46.4)
Group II	38 093 (19.1)	5277 (29.6)	700 (33.6)
Group III	16 063 (8.1)	1728 (9.7)	155 (7.5)
Group IV	8793 (4.41)	1937 (10.9)	261 (12.5)
**Comorbidities**			
M00-M99	46 886 (23.5)	7214 (40.4)	961 (46.2)
M00-M25	20 931 (10.5)	3214 (18.0)	587 (28.2)
M30-M36	1887 (1.0)	368 (2.1)	78 (3.8)
M40-M54	15 032 (7.5)	2764 (15.5)	367 (17.6)
M60-M79	22 978 (11.5)	4066 (22.8)	456 (21.9)
M80-M94	3520 (1.8)	544 (3.1)	88 (2.3)
M95-M99	1490 (0.8)	262 (1.5)	41 (2.0)
F00-F99	24 856 (12.5)	3665 (20.5)	416 (20.0)
F00-F09	2506 (1.3)	302 (1.7)	43 (2.1)
F10-F19	6184 (3.1)	708 (4.0)	105 (5.1)
F20-F29	2432 (1.2)	245 (1.4)	39 (1.9)
F30-F39	10 436 (5.2)	1521 (8.5)	175 (8.4)
F40-F48	14 613 (7.3)	2466 (13.8)	263 (12.6)
F50-F59	5656 (2.8)	791 (4.4)	117 (5.6)
F60-F69	4421 (2.2)	629 (3.5)	90 (4.3)
F70-F79	464 (0.2)	56 (0.3)	5 (0.2)
F80-F89	2155 (1.1)	270 (1.5)	51 (2.5)
F90-F98	4584 (2.3)	691 (3.9)	97 (4.6)
F99	3558 (1.8)	444 (2.5)	75 (3.6)

Other^a^ = Registered partner, Divorced partner, Surviving partner

Group I: No comorbidity present

Group II: MSD comorbidity present, but no MBD comorbidity

Group III: MBD comorbidity present, but no MSD comorbidity

Group IV: Both MSD and MBD comorbidity present

M00–M25: Arthropathies

M30–M36: Systemic connective tissue disorders

M40–M54: Dorsopathies

M60–M79: Soft tissue disorders

M80–M94: Osteopathies and chondropathies

M95–M99: Other disorders of the musculoskeletal system and connective tissue

F00–F99: Organic, including symptomatic, mental disorders

F10–F19: Mental and behavioural disorders due to psychoactive substance use

F20–F29: Schizophrenia, schizotypal and delusional disorders

F30–F39: Mood affective disorders

F40–F48: Neurotic, stress-related and somatoform disorders

F50–F59: Behavioural syndromes associated with physiological disturbances and physical factors

F60–F69: Disorders of adult personality and behaviour

F70–F79: Mental retardation

F80–F89: Disorders of psychological development

F90–F98: Behavioural and emotional disorders with onset usually occurring in childhood and adolescence

F99: Unspecified mental disorder

Table 3 shows the means of annual days on SL and DP during the entire 10-year follow-up period (-T5 to T5). In the group with no comorbidities (Group I), the TMD cohorts had two to three times more mean annual days of both SL and DP than the non-exposed cohort. For both MSD comorbidity (Group II) and MBD comorbidity (Group III), this difference was two-fold for SL. MSD (Group II) had a large impact on the 10-year annual difference in DP between the cohorts, whereas the group with MBD (Group III) did not display any major differences in DP between the exposed and the non-exposed. Table 3 also shows the mean annual days during the 10-year follow-up for each specific category of diagnoses of MSD and MBD. Unlike Group I–IV, these categories are not mutually excluded for other comorbidities. The TMD cohorts displayed higher dependence on both SL and DP for virtually all categories of diagnoses of MSD and MBD.

**Table 3:** Mean annual days of sick leave and disability pension in Groups I–IV as well as for individuals with diagnoses M00–M99 and F00–F99. Unlike Groups I–IV, specific diagnoses are not excluded for other comorbidities

	**Sick leave**						**Disability pension**					
	**NE**		**NS**		**S**		**NE**		**NS**		**S**	
	Mean	95% CI	Mean	95% CI	Mean	95% CI	Mean	95% CI	Mean	95% CI	Mean	95% CI
**Group**												
Group I	6.9	[6.9–7.0]	12.7	[12.4–13.0]	20.2	[18.9–21.4]	9.3	[9.2–9.4]	16.6	[16.2–17.1]	28.5	[26.7–17.1]
Group II	16.0	[15.8–16.2]	24.9	[24.3–25.5]	34.9	[33.0–36.8]	27.9	[27.6–28.2]	45.9	[44.9–46.9]	58.1	[55.3–60.9]
Group III	21.8	[21.5–22.2]	28.5	[27.3–29.7]	41.4	[36.7–46.0]	66.9	[66.2–67.6]	70.3	[68.2–72.3]	66.9	[60.4–73.6]
Group IV	35.1	[34.5–35.7]	43.4	[42.1–44.8]	49.0	[45.2–52.8]	86.7	[85.7–87.7]	107.3	[105.0–109.6]	114.7	[108.7–120.7]
**MSD**												
M00-M25	19.4	[19.2–19.7]	29.6	[28.7–30.4]	37.9	[35.7–40.0]	37.4	[36.9–37.8]	63.6	[62.2–65.1]	75.7	[72.3–79.1]
M30-M36	24.1	[23.1–25.1]	39.5	[36.5–42.5]	36.3	[30.4–42.1]	66.9	[65.1–68.8]	117.1	[111.9–122.3]	139.4	[128.3–150.5]
M40-M54	24.8	[24.4–25.1]	35.6	[34.6–36.7]	43.9	[40.1–46.9]	53.5	[52.9–54.1]	82.1	[80.4–83.8]	100.3	[95.4–105.1]
M60-M79	20.9	[20.6–21.2]	31.3	[30.5–32.1]	38.7	[36.1–41.2]	41.3	[40.8–41.7]	66.0	[64.7–67.3]	83.5	[79.4–87.6]
M80-M94	22.1	[21.4–22.8]	31.3	[29.2–33.5]	40.0	[34.2–45.8]	54.3	[53.0–55.6]	87.4	[83.5–91.4]	108.1	[97.8–118.4]
M95-M99	26.5	[25.3–27.8]	35.5	[32.2–38.7]	38.6	[30.5–46.6]	49.6	[47.6–51.5]	88.3	[82.5–94.2]	91.2	[77.4–105.03]
**MBD**												
F00-F09	22.3	[21.5–23.2]	32.2	[29.1–35.2]	39.0	[30.4–47.7]	165.6	[163.4–167.7]	156.9	[150.8–163.1]	163.3	[147.1–179.4]
F10-F19	24.1	[23.5–24.7]	35.9	[33.8–38.0]	47.0	[40.9–53.1]	85.1	[83.9–86.3]	101.9	[98.1–105.6]	119.4	[109.3–129.4]
F20-F29	20.9	[20.0–21.7]	35.4	[31.8–39.0]	44.4	[34.4–54.5]	178.0	[175.8–180.2]	160.1	[153.2–167.1]	158.6	[141.6–175.6]
F30-F39	34.5	[34.0–35.0]	44.3	[42.7–45.9]	50.2	[45.3–55.0]	90.2	[89.2–91.1]	109.2	[106.6–111.8]	112.8	[105.4–120.1]
F40-F48	29.2	[28.8–29.6]	38.0	[36.9–39.2]	47.5	[43.8–51.3]	78.6	[77.8–79.3]	93.4	[91.5–95.4]	101.5	[95.6–108.3]
F50-F59	23.1	[22.5–23.7]	32.3	[30.5–34.2]	40.0	[34.8–45.2]	100.6	[99.3–101.9]	123.3	[119.7–127.0]	142.6	[133.3–152.0]
F60-F69	28.6	[27.8–29.3]	36.8	[34.6–39.1]	46.3	[39.9–52.6]	126.7	[125.1–128.3]	156.6	[152.3–160.9]	134.5	[124.0–145.0]
F70-F79	5.3	[4.3–6.3]	8.7	[4.8–12.7]	22.7	[0.7–44.7]	282.5	[278.0–286.9]	275.0	[262.1–287.8]	258.7	[212.4–305.1]
F80-F89	18.4	[17.5–19.3]	30.1	[26.9–33.3]	34.9	[27.4–42.5]	145.4	[143.1–147.8]	151.4	[144.7–158.1]	183.8	[169.0–198.6]
F90-F98	26.6	[25.9–27.3]	34.0	[31.9–36.0]	52.8	[46.2–59.5]	106.0	[104.6–107.5]	132.0	[127.9–136.0]	145.4	[134.8–155.9]
F99	24.0	[23.3–24.8]	37.7	[35.0–40.3]	42.8	[35.9–49.7]	129.7	[127.9–131.4]	150.2	[145.2–155.2]	163.5	[151.4–175.5]

*MBD* Mental and behavioural disorders, *MSD* Diseases of the musculoskeletal and connective tissue, *NE* Non-exposed cohort, *NS* Non-surgical cohort, *S* Surgical cohort

M00–M25: Arthropathies

M30–M36: Systemic connective tissue disorders

M40–M54: Dorsopathies

M60–M79: Soft tissue disorders

M80–M94: Osteopathies and chondropathies

M95–M99: Other disorders of the musculoskeletal system and connective tissue

F00–F99: Organic, including symptomatic, mental disorders

F10–F19: Mental and behavioural disorders due to psychoactive substance use

F20–F29: Schizophrenia, schizotypal and delusional disorders

F30–F39: Mood affective disorders

F40–F48: Neurotic, stress-related and somatoform disorders

F50–F59: Behavioural syndromes associated with physiological disturbances and physical factors

F60–F69: Disorders of adult personality and behaviour

F70–F79: Mental retardation

F80–F89: Disorders of psychological development

F90–F98: Behavioural and emotional disorders with onset usually occurring in childhood and adolescence

F99: Unspecified mental disorder

**Fig. 3:**
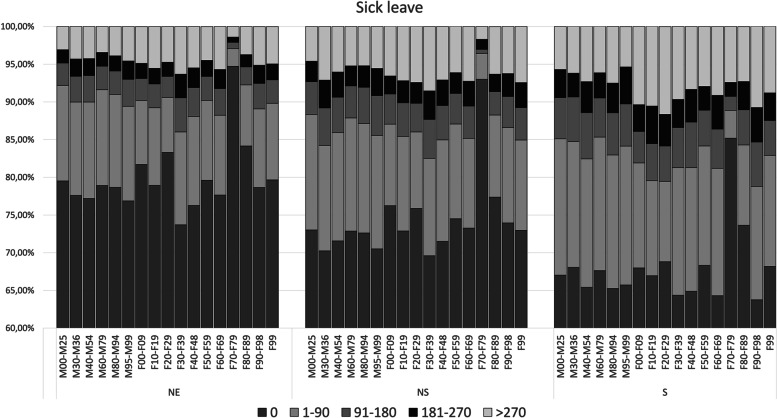
Mean annual days of SL during the 10-year follow-up (-T5 to T5) for each category of MSD and MBD, stratified on the three cohorts. Mean annual days are divided into five categories: 0, 1–90, 91–180, 181–270, and > 270 days (in accordance with how SL is granted). The diagnostic categories are not excluded for other co-existing comorbidities. M00–M25: Arthropathies, M30–M36: Systemic connective tissue disorders, M40–M54: Dorsopathies, M60–M79: Soft tissue disorders, M80–M94: Osteopathies and chondropathies, M95–M99: Other disorders of the musculoskeletal system and connective tissue, F00–F99: Organic, including symptomatic, mental disorders, F10–F19: Mental and behavioural disorders due to psychoactive substance use, F20–F29: Schizophrenia, schizotypal and delusional disorders, F30–F39: Mood affective disorders, F40–F48: Neurotic, stress-related and somatoform disorders, F50–F59: Behavioural syndromes associated with physiological disturbances and physical factors, F60–F69: Disorders of adult personality and behaviour, F70–F79: Mental retardation, F80–F89: Disorders of psychological development, F90–F98: Behavioural and emotional disorders with onset usually occurring in childhood and adolescence, F99: Unspecified mental disorder

**Fig. 4:**
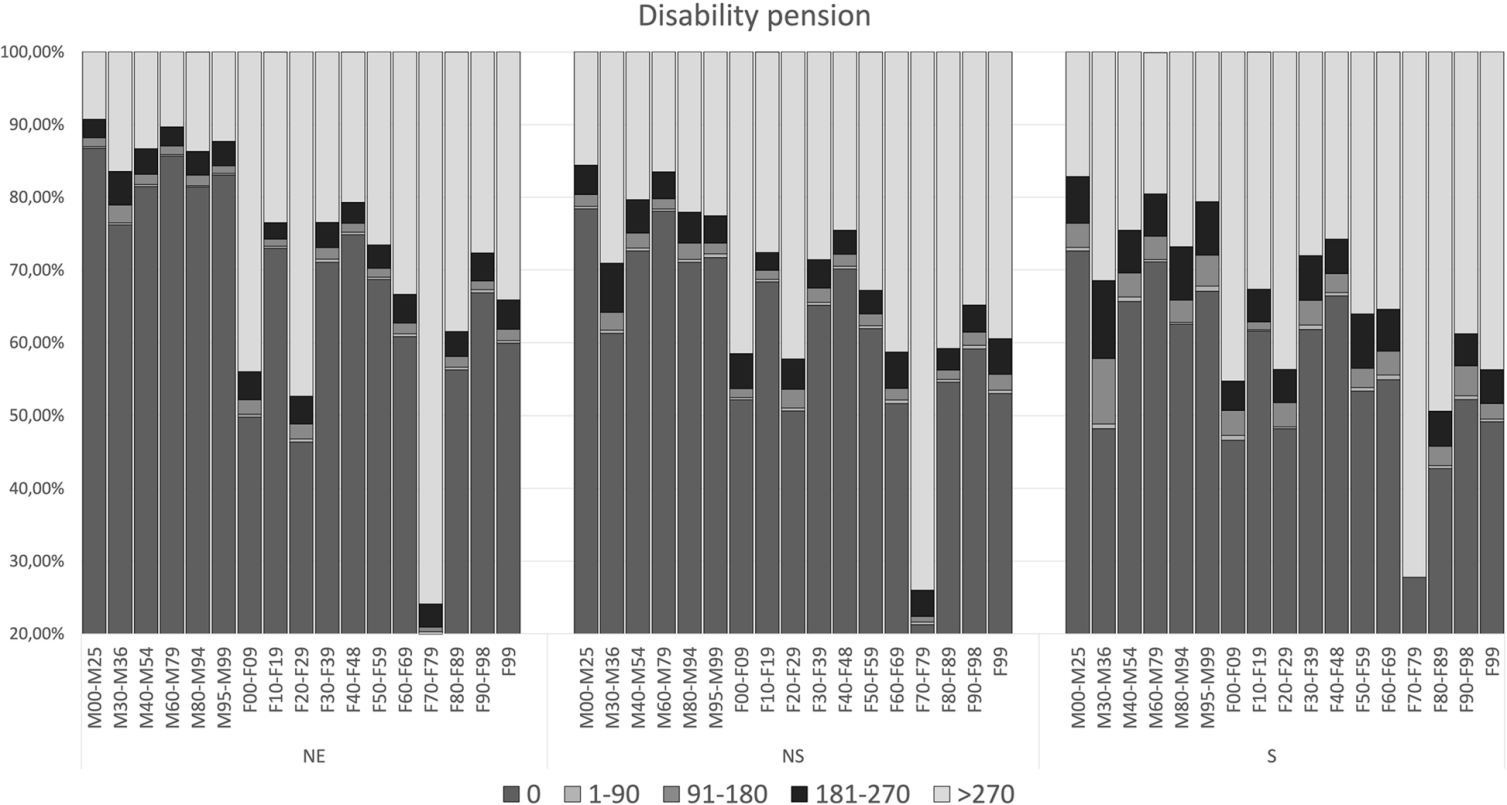
Mean annual days of DP during the 10-year follow-up (-T5 to T5) for each category of MSD and MBD, stratified on the three cohorts. Mean annual days are divided into five categories: 0, 1–90, 91–180, 181–270, and > 270 days (in accordance with how DP is granted). The diagnostic categories are not excluded for other co-existing comorbidities. M00–M25: Arthropathies, M30–M36: Systemic connective tissue disorders, M40–M54: Dorsopathies, M60–M79: Soft tissue disorders, M80–M94: Osteopathies and chondropathies, M95–M99: Other disorders of the musculoskeletal system and connective tissue, F00–F99: Organic, including symptomatic, mental disorders, F10–F19: Mental and behavioural disorders due to psychoactive substance use, F20–F29: Schizophrenia, schizotypal and delusional disorders, F30–F39: Mood affective disorders, F40–F48: Neurotic, stress-related and somatoform disorders, F50–F59: Behavioural syndromes associated with physiological disturbances and physical factors, F60–F69: Disorders of adult personality and behaviour, F70–F79: Mental retardation, F80-F89: Disorders of psychological development, F90–F98: Behavioural and emotional disorders with onset usually occurring in childhood and adolescence, F99: Unspecified mental disorder

Figure 3 shows the crude means of annual days of SL over the entire 10-year follow-up period, stratified for categories of diagnoses. The mean annual days are divided into categories of 0, 1–90, 91–180, 181–270, and > 270 days. The categories of diagnoses are not excluded for other concurrent comorbidities. Figure 4 depicts the corresponding means of DP from -T5 to T5.

### Main results

**Fig. 5:**
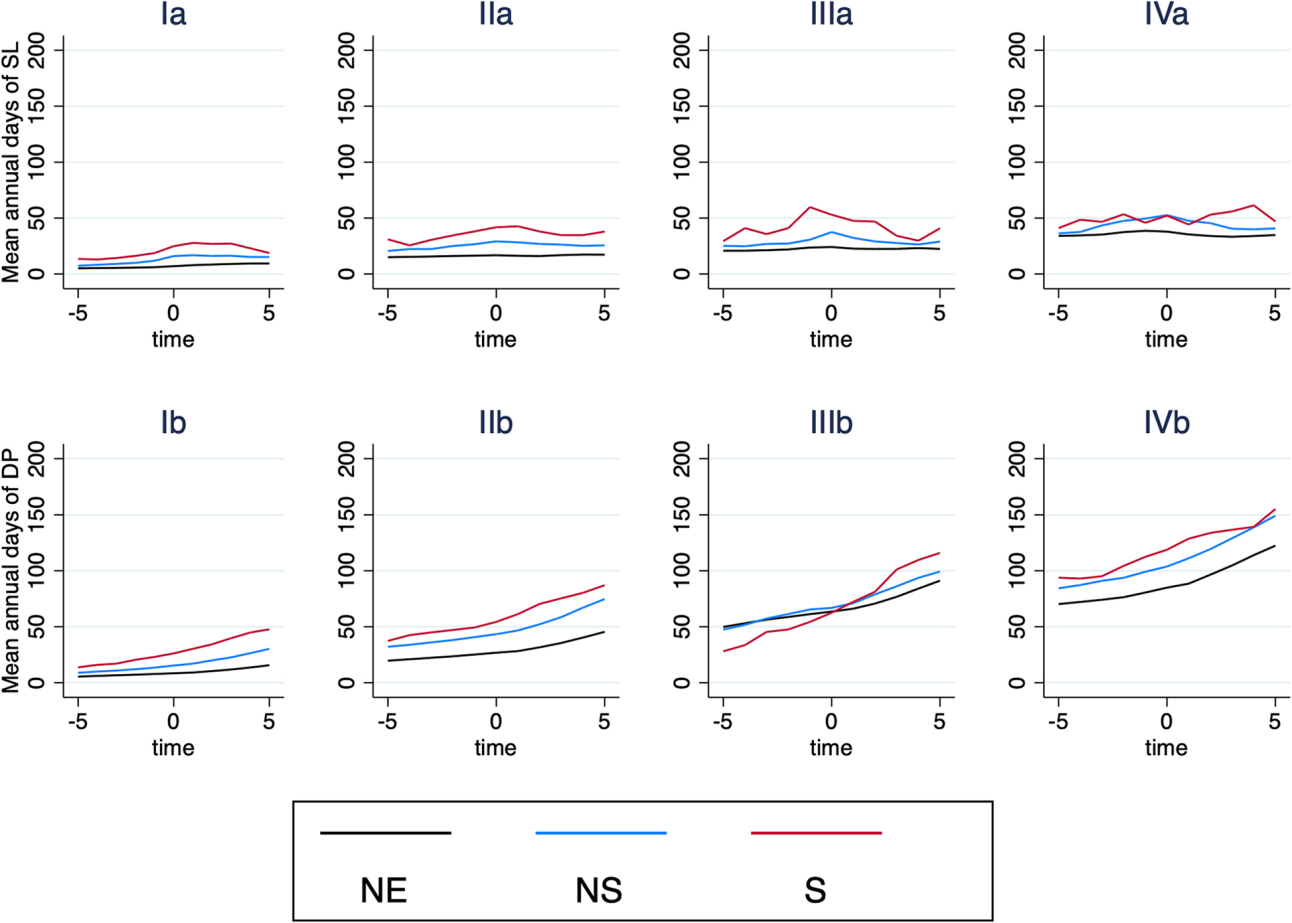
Predicted averages with 95% CI, drawn from generalized estimating equations and adjusted for all covariates. T0 is the time of inclusion. Ia: SL, no comorbidity (Group I), IIa: SL, MSD comorbidity (Group II), IIIa: SL, MBD comorbidity (Group III), IVa: SL, MSD and MBD comorbidity (Group IV), Ib: DP, no comorbidity (Group I), IIb: DP, MSD comorbidity (Group II), IIIb: DP, MBD comorbidity (Group III), IVb: DP, MSD and MBD comorbidity (Group IV), NE: Non-exposed cohort, NS: Non-surgical cohort, S: Surgical cohort

Results from the GEE regression model adjusted for age, sex, educational level, country of birth, DEGURBA, and year of inclusion for SL are shown in Table 4. The graphic output of the model is depicted in Fig. 5, graphs Ia–IVa. Significant differences in SL were found at all time points with a few exceptions. The differences in β-coefficients between the cohorts were substantially smaller in the group with both MSD and MBD (Group IV). Higher education (> 12 years) was associated with lower SL, with less impact in the MBD strata (Group III) and slightly higher impact on SL in the group with both MSD and MBD comorbidity (Group IV), compared to primary school (0–9 years), which was the reference. Being born outside of Europe was protective for all groups except for the MSD (Group II).

**Table 4:** Association between the cohorts and the outcome sick leave in relation to the prevalence of a comorbidity of musculoskeletal and connective tissue diseases and impaired mental health. The regression model was conducted with generalized estimating equations and adjusted for educational level, country of birth, and degree of urbanisation (DEGURBA). The interaction of time, sex and age is also displayed. Missing data in covariates are imputed with MICE (20 imputations)

Sick leave	Group I		Group II		Group III		Group IV	
	Graph Ia		Graph IIa		Graph IIIa		Graph IVa	
	β	95% CI	β	95% CI	β	95% CI	β	95% CI
**Cohort**								
General pop. cohort	0	(ref)	0	(ref)	0	(ref)	0	(ref)
Non-surgical	0.862	[0.856 0.868]	0.526	[0.520 0.532]	0.462	[0.453 0.470]	0.308	[0.301 0.315]
Surgical	1.009	[0.996 1.022]	0.743	[0.731 0.755]	0.723	[0.701 0.746]	0.333	[0.316 0.350]
**Time**								
-T5	-0.317	[-0.320 -.0314]	-0.109	[-0.112 -0.106]	-0.145	[-0.150 -0.141]	-0.109	[-0.113 -0.104]
-T4	-0.275	[-0.278 -0.272]	-0.091	[-0.095 -0.088]	-0.147	[-0.151 -0.143]	-0.093	[-0.098 -0.089]
-T3	-0.246	[-0.249 -0.243]	-0.070	[-0.073 -0.066]	-0.128	[-0.132 -0.124]	-0.075	[-0.079 -0.071]
-T2	-0.195	[-0.197 -0.192]	-0.037	[-0.040 -0.034]	-0.093	[-0.097 -0.090]	-0.013	[-0.016 -0.009]
-T1	-0.134	[-0.136 -0.132]	-0.022	[-0.025 -0.020]	-0.019	[-0.022 -0.016]	0.020	[0.017 0.023]
T0	0	(ref)	0	(ref)	0	(ref)	0	(ref)
T1	0.124	[0.122 .0126]	-0.031	[-0.033 -0.028]	-0.062	[-0.065 -0.059]	-0.069	[-0.072 -0.066]
T2	0.156	[0.153 0.158]	-0.09	[-0.093 -0.087]	-0.105	[-0.109 -0.101]	-0.122	[-0.126 -0.118]
T3	0.176	[0.173 0.178]	-0.093	[-0.096 -0.089]	-0.131	[-0.135 -0.126]	-0.158	[-0.163 -0.153]
T4	0.172	[0.169 0.175]	-0.134	[-0.138 -0.131]	-0.148	[-0.153 -0.144]	-0.173	[-0.179 -0.168]
T5	0.119	[0.116 0.122]	-0.211	[-0.215 -0.206]	-0.214	[-0.220 -0.209]	-0.189	[-0.195 -0.183]
Time*Non-surgical								
-T5	-0.465	[-0.475 -0.455]	-0.246	[-0.255 -0.238]	-0.255	[-0.267 -0.242]	-0.268	[-0.278 -0.257]
-T4	-0.397	[-0.406 -0.388]	-0.183	[-0.191 -0.175]	-0.267	[-0.279 -0.255]	-0.244	[-0.254 -0.234]
-T3	-0.328	[-0.337 -0.320]	-0.206	[-0.213 -0.198]	-0.209	[-0.220 -0.197]	-0.118	[-0.127 -0.109]
-T2	-0.284	[-0.292 -0.276]	-0.121	[-0.128 -0.115]	-0.227	[-0.237 -0.217]	-0.091	[-0.099 -0.083]
-T1	-0.166	[-0.172 -0.160]	-0.069	[-0.074 -0.063]	-0.185	[-0.193 -0.177]	-0.082	[-0.089 -0.076]
T0	0	(ref)	0	(ref)	0	(ref)	0	(ref)
T1	-0.077	[-0.083 -0.072]	-0.001	[-0.007 0.004]*	-0.081	[-0.089 -0.074]	-0.031	[-0.037 -0.025]
T2	-0.182	[-0.189 -0.175]	-0.037	[-0.043 -0.030]	-0.175	[-0.185 -0.164]	-0.038	[-0.046 -0.029]
T3	-0.230	[-0.237 -0.222]	-0.105	[-0.113 -0.097]	-0.228	[-0.241 -0.216]	-0.140	[-0.151 -0.130]
T4	-0.353	[-0.362 -0.345]	-0.175	[-0.184 -0.167]	-0.300	[-0.313 -0.286]	-0.180	[-0.192 -0.169]
T5	-0.357	[-0.365 -0.348]	-0.151	[-0.161 -0.142]	-0.171	[-0.186 -0.157]	-0.188	[-0.202 -0.175]
Time*Surgical								
-T5	-0.297	[-0.319 -0.275]	-0.191	[-0.209 -0.173]	-0.447	[-0.483 -0.411]	-0.144	[-0.169 -0.120]
-T4	-0.377	[-0.398 -0.356]	-0.399	[-0.417 -0.381]	-0.112	[-0.144 -0.081]	0.007	[-0.015 0.030]*
-T3	-0.311	[-0.331 -0.291]	-0.245	[-0.261 -0.229]	-0.271	[-0.301 -0.240]	-0.040	[-0.061 -0.018]
-T2	-0.238	[-0.255 -0.220]	-0.153	[-0.168 -0.139]	-0.166	[-0.193 -0.140]	0.049	[0.030 0.067]
-T1	-0.154	[-0.168 -0.141]	-0.070	[-0.081 -0.059]	0.137	[0.118 0.157]	-0.142	[-0.157 -0.127]
T0	0	(ref)	0	(ref)	0	(ref)	0	(ref)
T1	-0.013	[-0.026 -0.001]	0.051	[0.040 0.062]	-0.043	[-0.064 -0.023]	-0.099	[-0.114 -0.084]
T2	-0.089	[-0.104 -0.073]	-0.025	[-0.039 -0.011]	-0.033	[-0.059 -0.006]	0.124	[0.105 0.143]
T3	-0.117	[-0.134 -0.100]	-0.127	[-0.143 -0.111]	-0.339	[-0.372 -0.307]	0.179	[0.157 0.201]
T4	-0.301	[-0.319 -0.283]	-0.129	[-0.146 -0.111]	-0.481	[-0.518 -0.444]	0.279	[0.256 0.303]
T5	-0.484	[-0.505 -0.464]	-0.010	[-0.028 0.008]*	-0.118	[-0.153 -0.082]	-0.023	[-0.051 0.005]*
**Sex**								
Male	0	(ref)	0	(ref)	0	(ref)	0	(ref)
Female	0.697	[0.694 0.699]	0.592	[0.589 0.595]	0.520	[0.516 0.525]	0.321	[0.316 0.326]
**Educational level**								
0–9 years	0	(ref)	0	(ref)	0	(ref)	0	(ref)
10–12 years	-0.090	[-0.093 -0.086]	-0.088	[-0.092 -0.084]	0.163	[0.156 0.169]	0.167	[0.160 0.174]
> 12 years	-0.506	[-0.510 -0.502]	-0.531	[-0.535 -0.526]	-0.028	[-0.035 0.021]	0.039	[0.029 0.048]
**Country of birth**								
Sweden	0	(ref)	0	(ref)	0	(ref)	0	(ref)
Nordic countries	0.015	[0.009 0.020]	-0.001	[-0.007 0.006]*	0.116	[0.106 0.125]	-0.030	[-0.041 -0.020]
European countries	0.144	[0.140 0.147]	0.179	[0.174 0.183]	-0.043	[-0.050 -0.036]	0.110	[0.103 0.116]
Outside Europe	-0.216	[-0.220 -0.212]	0.091	[0.086 0.095]	-0.297	[-0.304 -0.291]	-0.051	[-0.057 -0.044]
**DEGURBA**								
Cities	0	(ref)	0	(ref)	0	(ref)	0	(ref)
Towns and suburbs	0.096	[0.092 0.099]	0.136	[0.132 0.140]	0.097	[0.092 0.102]	0.102	[0.095 0.108]
Rural areas	0.048	[0.045 0.052]	0.147	[0.143 0.151]	0.042	[0.037 0.048]	0.089	[0.082 0.095]
**Age**								
23–25 (ref)	0	(ref)	0	(ref)	0	(ref)	0	(ref)
26–30	0.420	[0.414 0.427]	0.644	[0.633 0.655]	0.667	[0.656 0.678]	0.666	[0.651 0.682]
31–35	0.705	[0.698 0.711]	0.944	[0.933 0.954]	1.085	[1.075 1.096]	1.039	[1.025 1.054]
36–40	0.850	[0.844 0.856]	1.033	[1.023 1.043]	1.236	[1.225 1.246]	1.274	[1.260 1.288]
41–45	0.918	[0.912 0.924]	1.071	[1.061 1.081]	1.207	[1.196 1.217]	1.212	[1.198 1.226]
46–50	0.947	[0.941 0.952]	1.087	[1.077 1.097]	1.188	[1.178 1.199]	1.192	[1.178 1.206]
51–55	1.077	[1.071 1.083]	1.153	[1.143 1.163]	1.094	[1.083 1.105]	1.075	[1.061 1.089]
≥ 56	1.126	[1.120 1.132]	1.126	[1.116 1.136]	0.999	[0.988 1.010]	1.053	[1.039 1.067]

Group I: No comorbidity present

Group II: MSD comorbidity present, but no MBD comorbidity

Group III: MBD comorbidity present, but no MSD comorbidity

Group IV: Both MSD and MBD comorbidity present

^*^p > 0.05

For DP, the results from the GEE regression model adjusted for the same covariates are shown in Table 5, and the graphic output is displayed in Fig. 5, graphs Ib–IVb. Significant differences between the cohorts in annual DP days were found at most time points. The TMD cohorts had higher number of mean annual days of DP in all groups except for the MBD group (Group III), where the surgical cohort had a negative β-coefficient. Higher education had slightly less impact on DP in the group with both MSD and MBD (Group IV) than for the other groups. Moreover, whereas male sex was less associated with DP for Groups I, II, and IV, the difference in sex was substantially smaller in Group III. In Group III and IV, higher age had less impact on DP days than for the other groups.

**Table 5:** Association between the cohorts and the outcome disability pension in relation to the prevalence of a comorbidity of musculoskeletal and connective tissue diseases and impaired mental health. The regression model was conducted with generalized estimating equations and adjusted for educational level, country of birth, and degree of urbanisation (DEGURBA). The interaction of time, sex, and age is also displayed. Missing data in covariates are imputed with MICE (20 imputations)

Disability pension	Group I		Group II		Group III		Group IV	
	Graph Ib		Graph IIb		Graph IIIb		Graph IVb	
	β	95% CI	β	95% CI	β	95% CI	β	95% CI
**Cohort**								
General pop. cohort	0	(ref)	0	(ref)	0	(ref)	0	(ref)
Non-surgical	0.596	[0.590 0.601]	0.443	[0.439 0.448]	0.072	[0.066 0.079]	0.206	[0.201 0.211]
Surgical	0.906	[0.893 0.919]	0.617	[0.606 0.628]	-0.029	[-0.050 -0.009]	0.366	[0.354 0.378]
**Time**								
-T5	-0.403	[-0.404 -0.402]	-0.306	[-0.307 -0.304]	-0.238	[-0.240 -0.237]	-0.188	[-0.189 -0.186]
-T4	-0.315	[-0.316 -0.314]	-0.244	[-0.245 -0.243]	-0.173	[-0.174 -0.172]	-0.160	[-0.162 -0.159]
-T3	-0.226	[-0.227 -0.225]	-0.183	[-0.184 -0.182]	-0.113	[-0.115 -0.112]	-0.133	[-0.134 -0.132]
-T2	-0.152	[-0.153 -0.152]	-0.127	[-0.128 -0.126]	-0.075	[-0.076 -0.074]	-0.104	[-0.105 -0.103]
-T1	-0.071	[-0.072 -0.071]	-0.062	[-0.063 -0.061]	-0.035	[-0.035 -0.034]	-0.052	[-0.053 -0.051]
T0	0	(ref)	0	(ref)	0	(ref)	0	
T1	0.077	[0.076 0.078]	0.050	[0.050 0.051]	0.039	[0.038 0.039]	0.040	[0.039 0.041]
T2	0.150	[0.150 0.151]	0.097	[0.096 0.097]	0.065	[0.064 0.066]	0.073	[0.071 0.074]
T3	0.222	[0.221 0.223]	0.135	[0.134 0.136]	0.093	[0.092 0.094]	0.096	[0.095 0.098]
T4	0.283	[0.282 0.285]	0.161	[0.160 0.163]	0.113	[0.111 0.114]	0.122	[0.120 0.123]
T5	0.343	[0.342 0.344]	0.188	[0.186 0.189]	0.135	[0.133 0.136]	0.146	[0.144 0.148]
Time*Non-surgical								
-T5	-0.134	[-0.138 -0.129]	0.005	[0.001 0.008]	-0.094	[-0.099 -0.089]	-0.027	[-0.031 -0.023]
-T4	-0.115	[-0.119 -0.111]	-0.005	[-0.008 -0.002]	-0.074	[-0.078 -0.070]	-0.013	[-0.017 -0.010]
-T3	-0.115	[-0.118 -0.111]	-0.005	[-0.008 -0.003]	-0.034	[-0.037 -0.030]	0.004	[0.001 0.007]
-T2	-0.082	[-0.085 -0.079]	-0.004	[-0.006 -0.002]	-0.005	[-0.008 -0.002]	0.004	[0.001 0.007]
-T1	-0.049	[-0.051 -0.047]	-0.001	[-0.003 0.000]*	0.015	[0.013 0.017]	0.008	[0.006 0.010]
T0	0	(ref)	0	(ref)	0	(ref)	0	
T1	0.031	[0.029 0.032]	0.022	[0.021 0.024]	0.019	[0.017 0.021]	0.027	[0.025 0.029]
T2	0.058	[0.056 0.061]	0.026	[0.024 0.028]	0.049	[0.046 0.052]	0.019	[0.016 0.021]
T3	0.055	[0.052 0.058]	0.036	[0.033 0.038]	0.060	[0.056 0.063]	0.014	[0.011 0.018]
T4	0.067	[0.064 0.071]	0.048	[0.045 0.051]	0.070	[0.066 0.074]	0.007	[0.004 0.011]
T5	0.068	[0.065 0.072]	0.048	[0.045 0.051]	0.053	[0.049 0.058]	0.008	[0.004 0.013]
Time*Surgical								
-T5	-0.225	[-0.236 -0.214]	-0.065	[-0.073 -0.057]	-0.518	[-0.538 -0.498]	-0.050	[-0.059 -0.041]
-T4	-0.171	[-0.180 -0.162]	0.001	[-0.006 0.008]*	-0.418	[-0.435 -0.402]	-0.085	[-0.094 -0.077]
-T3	-0.198	[-0.206 -0.190]	-0.005	[-0.011 0.001]*	-0.194	[-0.206 -0.181]	-0.090	[-0.098 -0.083]
-T2	-0.094	[-0.100 -0.088]	-0.014	[-0.019 -0.010]	-0.189	[-0.199 -0.178]	-0.027	[-0.033 -0.021]
-T1	-0.061	[-0.065 -0.057]	-0.034	[-0.038 -0.031]	-0.101	[-0.108 -0.094]	-0.004	[-0.008 0.000]
T0	0	(ref)	0	(ref)	0	(ref)	0	
T1	0.071	[0.067 0.075]	0.069	[0.066 0.072]	0.104	[0.097 0.111]	0.041	[0.037 0.045]
T2	0.100	[0.095 0.106]	0.133	[0.128 0.137]	0.174	[0.165 0.184]	0.010	[0.004 0.015]
T3	0.152	[0.146 0.158]	0.146	[0.141 0.152]	0.329	[0.317 0.340]	-0.048	[-0.055 -0.041]
T4	0.179	[0.172 0.186]	0.131	[0.125 0.137]	0.351	[0.338 0.364]	-0.069	[-0.078 -0.061]
T5	0.141	[0.134 0.149]	0.137	[0.130 0.144]	0.336	[0.322 0.350]	-0.049	[-0.058 -0.040]
**Sex**								
Male	0	(ref)	0	(ref)	0	(ref)	0	(ref)
Female	0.525	[0.521 0.529]	0.642	[0.638 0.646]	0.033	[0.030 0.037]	0.220	[0.216 0.225]
**Educational level**								
0–9 years	0	(ref)	0	(ref)	0	(ref)	0	(ref)
10–12 years	-0.750	[-0.749 -0.742]	-0.444	[-0.451 -0.438]	-0.503	[-0.514 -0.492]	-0.372	[-0.382 -0.363]
> 12 years	-1.689	[-1.719 -1.658]	-1.452	[-1.468 -1.436]	-1.237	[-1.253 -1.222]	-0.947	[-0.971 -0.922]
**Country of birth**								
Sweden	0	(ref)	0	(ref)	0	(ref)	0	(ref)
Nordic countries	0.281	[0.274 0.289]	0.228	[0.221 0.235]	0.043	[0.034 0.052]	0.097	[0.087 0.107]
European countries	0.592	[0.586 0.598]	0.576	[0.569 0.582]	0.163	[0.156 0.170]	0.168	[0.161 0.175]
Outside Europe	0.130	[0.122 0.137]	0.282	[0.276 0.289]	-0.188	[-0.194 -0.181]	-0.025	[-0.031 -0.019]
**DEGURBA**								
Cities	0	(ref)	0	(ref)	0	(ref)	0	(ref)
Towns and suburbs	0.036	[0.031 0.041]	0.112	[0.106 0.118]	0.080	[0.073 0.087]	0.138	[0.131 0.146]
Rural areas	0.051	[0.045 0.056]	0.145	[0.139 0.152]	0.098	[0.092 0.104]	0.119	[0.112 0.126]
**Age**								
23–25 (ref)	0	(ref)	0	(ref)	0	(ref)	0	(ref)
26–30	-0.312	[-0.326 -0.298]	0.171	[0.150 0.193]	-0.082	[-0.093 -0.072]	-0.013	[-0.029 0.002]*
31–35	0.136	[0.124 0.148]	0.587	[0.568 0.607]	0.002	[-0.008 0.012]*	0.127	[0.112 0.142]
36–40	0.498	[0.486 0.509]	0.904	[0.885 0.923]	0.353	[0.343 0.362]	0.466	[0.451 0.480]
41–45	0.872	[0.861 0.883]	1.311	[1.293 1.329]	0.625	[0.616 0.634]	0.756	[0.742 0.769]
46–50	1.282	[1.271 1.292]	1.642	[1.624 1.660]	0.910	[0.901 0.919]	0.999	[0.986 1.012]
51–55	1.718	[1.707 1.728]	1.978	[1.960 1.995]	1.089	[1.080 1.097]	1.233	[1.220 1.246]
≥ 56	2.086	[2.075 2.097]	2.272	[2.254 2.290]	1.247	[1.238 1.255]	1.392	[1.379 1.405]

Group I: No comorbidity present

Group II: MSD comorbidity present, but no MBD comorbidity

Group III: MBD comorbidity present, but no MSD comorbidity

Group IV: Both MSD and MBD comorbidity present

^*^p > 0.05

## Discussion

In this study, we show that the higher dependence on SL and DP benefits among TMD patients compared to the general population remains even when considering MSD and MBD comorbidities. Irrespective of comorbidity, the TMD subjects had more mean annual days of SL and DP than the general population. There were some exceptions for certain MBD diagnoses such as F00–F09 (organic, including symptomatic, and mental disorders), F20–F29 (schizophrenia, schizotypal, and delusional disorders), and F70–F79 (mental retardation). That is, the non-exposed cohort had more annual days of DP than the exposed cohorts. The findings are important as they emphasize how work incapacity of TMD patients is strongly influenced by comorbidities and that the higher dependence on SL and DP benefits compared to the general population in Group I is most likely attributed to TMD. Therefore, the increased need for social insurance benefits among TMD patients cannot be solely explained by comorbidities.

A previous SWEREG-TMD study showed that TMD patients are 2–3 times more reliant on SL and DP benefits than the general population, in particular patients with TMD that requires multiple surgical interventions [12]. TMD related to the joint that requires repeated surgery is usually associated with inflammatory and degenerative disorders, and synovial fluid from patients have shown increased levels of inflammatory mediators [19]. These patients often have arthritis, osteoarthritis, psoriatic arthritis, rheumatoid arthritis, and ankylosing spondylitis, and the most severe cases may even need complete joint removal and replacement with a prosthesis [20]. Considering these known underlying systemic diseases and the association they have to SL and DP, it was of utmost importance to integrate underlying diseases in a study of SL and DP for TMD patients [8, 21].

In Sweden and the other Nordic countries, the healthcare system is mainly tax financed, so patients pay a very small fee to receive treatment. However, as the dental care system is tax financed to a considerably lesser extent, dental patients have higher out-of-pocket expenses [22]. Patients with painful TMD seek help both from the health care services and from the dental care service. Management sometimes falls between dentists and general practitioners (GPs) since there is uncertainty about who is responsible for the patient – the dentist or the GP [23], the longer the pain remains, the harder it is to diagnose. Dental and medical record systems are currently not compatible in Northern Europe, which makes it difficult for professionals to collaborate in the management of the patients. In addition to expenses associated with dental treatment, avoidance due to fear or anxiety may also explain why some people do not seek professional dental care when needed [24–26]. Considering the increased societal costs associated with patients with TMD found in this study, it is important to address whether earlier prevention or treatment programs for patients with certain underlying diseases with an increased risk for TMD would decrease a patient’s incapacity to work and consequently reduce suffering and societal costs.

In a study focused on SL among patients with MSD, a large heterogeneity was seen between the different types of diagnoses, with the lowest number of SL days during a two-year period (2009–2010) for low back pain and myalgia and the highest in disc disorders and rheumatoid arthritis [21]. The authors of the study found large differences in the need of SL and therefore advised against grouping MSD diagnoses together, an approach applied in this study. Although it would be of great interest to look specifically at SL and DP by specific diagnoses, those results would be too extensive to present within the scope of this study. This focus, however, may be presented in future publications.

In this study, more comorbidities among TMD patients resulted in more days of SL and DP. This finding is not surprising and multiple comorbidities have been shown to affect the trajectory and need of social insurance benefits for other conditions as well [27–31]. Our results also show increased reliance on social insurance benefits even before diagnosis and inclusion in the study. This finding might be explained by late diagnosis, comorbidities, or diagnosis and treatment outside of the health care system. A similar pattern was seen in patients with Crohn’s disease, where the patients received SL and DP benefits long before diagnosis, which could be explained by delayed diagnosis or comorbidities [32]. Common conditions such as attention-deficit/hyperactivity disorder (ADHD) have also been shown to increase the need of SL and DP, where comorbid conditions could explain one-third of the link between ADHD and DP [33]. SL, DP, and unemployment in patients who have depressive anxiety and stress-related disorders are also highly affected by other mental disorder comorbidities, suggesting an additive effect [34]. Such additive effects may partially explain what is seen in the group with both MSD and MBD comorbidity (Group IV) in this study: SL and DP are higher in this group than in the other groups, but the difference between the general population and the TMD cohorts is diminishing.

There is an intricate nexus of causal exposures that impact the need for SL and DP among any patient groups, including TMD patients. The impact of comorbidities, such as psychiatric-somatic diagnoses, on SL and DP and the ability to return to work have been established for many diseases [35–38]. Psychological factors in particular have been proposed as important mediators for the need of SL in patients with musculoskeletal pain, a finding that is also seen in this study [39]. The need of DP among patients with MSD is suggested to be partially driven by mental health, and the period after a DP spell might even increase the risk of impaired mental health, creating a possible causal nexus [40]. It has also been established that individuals who receive DP with a depressive disorder have an increased risk of suicidal behaviour [41]. As TMD is also associated with increased risk for suicidal behaviour, it is important to recognize that this patient group is not only extremely vulnerable and in need of social insurance benefits but also that combined comorbidities increase the risk of even more serious adverse outcomes [42, 43]. Therefore, patient-specific examination, treatment, and follow-up is of utmost importance for these vulnerable patients.

### Strengths and limitations

The most substantial strength of this study is the prospective nature of registry-collected data with no loss to follow-up, which the results are based on. The Swedish national registries contain high quality data that have high validity. As the number of subjects included in this study is unique, this may be the first study to investigate the impact of comorbidities on the use of social insurance benefits among TMD patients.

The largest limitation of this study is that TMD represents a heterogenic patient group that in Sweden is primarily treated within the dental care system and therefore not available in the registries used in this study. Therefore, the non-surgical cohort represents a subgroup of all TMD patients who do not require surgical treatment for their condition, and the conclusions drawn about this subgroup may not be applicable to all TMD patients. Therefore, future studies should investigate how patients who are conservatively treated within the dental care system differ from the TMD patients diagnosed but not treated within the health care system. Furthermore, it is not possible to rule out that patients within the non-surgical cohort need surgical treatment later, causing a possible misclassification bias, specifically for subjects included late in the study. This study does not address whether earlier intervention, treatment, or prevention of severe TMD increases the patient’s ability to work, so studies on the matter are warranted. It is also important to address that the results might be less relevant in countries where private health insurances are used to a larger extent; nevertheless, the results indicate the burden of disease the TMD patients carry, specifically patients with several comorbidities.

## Conclusion

TMD patients were more dependent on SL and DP benefits compared to general population and the difference remained even after stratifying MSD and MBD comorbidity. In individuals with combined MSD and MBD comorbidity, concurrent TMD had less impact on the need for social insurance benefits. The results accentuate TMD patients’ impaired ability to return to work, specifically in the light of common comorbidities, and emphasize why TMD should be recognized as a disorder with a potential to substantially impact individual and economic suffering and societal costs.

## Data Availability

The data that support the findings of this study are available from Swedish NBHW and Statistics Sweden, but restrictions apply to the availability of these data, which were used under license for the current study, and so are not publicly available. Data are however available from the corresponding author ASF, upon reasonable request and with permission of the Swedish NBHW, Statistics Sweden and the Swedish Ethical Review Authority.

## Abbreviations

DP: Disability pension
DEGURBA: Eurostat’s Degree of Urbanisation
ICD-10: International Classification of Diseases (10^th^ revision)
LISA: The Longitudinal Integrated Database for Labour Market and Health Insurance Studies
MBD: Mental and Behavioural Disorders
MSD: Diseases of the Musculoskeletal System and Connective Tissue
NBHW: National Board of Health and Welfare
NE: Non-exposed cohort
NS: Non-surgical cohort
NPR: National Patient Registry
PIN: Personal Identification Number
S: Surgical cohort
SCB: Statistics Sweden
SDI: Sociodemographic index
SL: Sick leave
SWEREG-TMD: Swedish National Registry Studies for Surgically Treated TMD
TMD: Temporomandibular Disorders
TMJ: Temporomandibular Joint
TPR: Total Population Registry
YLD: Years Lived with Disability
M00–M25: Arthropathies
M30–M36: Systemic connective tissue disorders
M40–M54: Dorsopathies
M60–M79: Soft tissue disorders
M80–-M94: Osteopathies and chondropathies
M95–M99: Other disorders of the musculoskeletal system and connective tissue
F00–F99: Organic, including symptomatic, mental disorders
F10–F19: Mental and behavioural disorders due to psychoactive substance use
F20–F29: Schizophrenia, schizotypal and delusional disorders
F30–F39: Mood affective disorders
F40–F48: Neurotic, stress-related and somatoform disorders
F50–F59: Behavioural syndromes associated with physiological disturbances and physical factors
F60–F69: Disorders of adult personality and behaviour
F70–F79: Mental retardation
F80–F89: Disorders of psychological development
F90–F98: Behavioural and emotional disorders with onset usually occurring in childhood and adolescence
F99: Unspecified mental disorder
